# Mutagenic, Genotoxic and Immunomodulatory effects of Hydroxychloroquine and Chloroquine: a review to evaluate its potential to use as a prophylactic drug against COVID-19

**DOI:** 10.1186/s41021-020-00164-0

**Published:** 2020-09-02

**Authors:** Allan Giri, Ankita Das, Ajoy K. Sarkar, Ashok K. Giri

**Affiliations:** 1 Department of Biomedical Science, Kansas City University of Medicine and Biosciences, Kansas City, MO 64106 USA; 2 Department of Environmental Sciences, University of Calcutta, Kolkata, 700019 India; 3 Intensive Care Unit, Peerless Hospital, B.K. Roy Research Centre, Kolkata, 700094 India; 4 Molecular Genetics Division, CSIR-Indian Institute of Chemical Biology, 4 Raja S. C. Mullick Road, Kolkata, 700032 India

**Keywords:** Hydroxychloroquine, Chloroquine, Mutagenicity, Genotoxicity, Immunomodulation, prophylactic use, COVID-19

## Abstract

Hydroxychloroquine (HCQ) and Chloroquine (CQ) are two anti-malarial drugs that are now being extensively used by front-line healthcare workers and other common people as a prophylactic drug against the Corona Virus Disease − 19 (COVID-19) in India and as well as in many parts of the world. While only a few in vitro studies have pointed to some efficacy of these drugs as a prophylactic against COVID-19, to date, there are no clinical studies that have established any clinical efficacy of these drugs as a prophylactic. These drugs are commonly used for the treatment of Rheumatoid Arthritis (RA) and Systemic Lupus Erythematosus (SLE) because of its immunomodulatory effects. Previously, we have evaluated the genetic toxicology of different drugs and chemicals including antimalarial drug CQ both in vitro and in vivo. Thus, we recognize the need to critically review the mutagenic, genotoxic, and immunomodulatory effects of these drugs, to find out whether it is safe to use as a prophylactic drug against COVID-19. Existing literature suggests that CQ can induce mutagenic and genotoxic effects in multiple test systems and both the drugs have immunomodulatory effects. There was no data available to evaluate the mutagenicity and genotoxicity for HCQ. However, during metabolism about 60% of both the drugs remain unchanged and about 40% of the drugs are metabolized into two metabolites, desethylchloroquine and bisdesethylchloroquine by the action of the cytochrome P450 (CYP) enzymes in the liver. Both HCQ and CQ are immunomodulatory drugs and have the potential to suppress normal immune system activation. In this review, we have elucidated the mechanism of immunomodulation by both HCQ and CQ and highlighted the mutagenic and genotoxic effects from the available literature. This article is written with the sole objective that the reader will be able to recognize the adverse effects of these drugs when consumed by healthy individuals as a prophylactic. Current literature indicates that healthy individuals should refrain from the use of these drugs until further investigation.

## Background

The novel Severe Acute Respiratory Syndrome Corona Virus 2 (COVID-19) pandemic has now become a nightmare throughout the world. This pandemic has caused serious health crisis not only among the poor nations but also across the world’s advanced countries. Researches all across the globe are trying to find an effective drug that would show promising results to prevent or to treat and control the COVID- 19. Recently, scientists have pointed out that the novel COVID-19 was transmissible in aerosol [1]. Thus, it puts the healthcare workers at risk who works in close proximity with COVID-19 patients. This demanded the need for a prophylactic drug against COVID-19 amongst healthcare workers.

Hydroxychloroquine (HCQ) and Chloroquine (CQ) are two antimalarial drugs that remain the universally accepted drugs for the treatment of Rheumatoid arthritis and Systemic Lupus Erythematosus [2–5]. These two drugs have shown some results in inhibiting the novel COVID-19 in vitro [6, 7]. A recent study demonstrated that certain cell types when treated with HCQ or CQ and then exposed to the novel COVID-19 strain, presented antiviral activity and that HCQ was more potent than CQ [8]. On the other hand, another in vitro study found out that CQ was potent than HCQ at all four different multiplicities of infection to act as antiviral when exposed to COVID-19 post-incubation with these drugs [7]. Additionally, CQ was able to act as anti-viral both pre and post-infection against the COVID-19 in vitro [6]. These findings may have led to the proposal and optimistic use of HCQ and CQ as prophylactics.

Yet historically, we have seen that in vitro studies don’t always translate in vivo or human subjects. For instance, despite the strong evidence of CQ as a prophylactic against influenza A and B in vitro, CQ was not effective to prevent either influenza A or B in the human subjects [9]. Rather, dizziness, nausea, and diarrhea were more common in the CQ group compared to the control (placebo) group. Another study showed that Ebola virus replication was successfully inhibited in vitro by CQ, however, it failed in guinea pig models in vivo [10]. Yet another study showed CQ enhanced Chikungunya virus replication in vivo when in fact CQ had been shown to have an effective inhibitory effect in vitro [11]. Thus, to date, with the lack of any controlled clinical trials, the clinical effectiveness of these drugs as prophylactics against COVID-19 in vivo remains unanswered.

Like any other drugs, these drugs also comes with certain risks. So, we mustn’t overlook the toxicological risks while making a rational decision of using these drugs as prophylactics. Previously, we have extensively reviewed and evaluated the genetic toxicology of antimalarial, analgesics, antipyretic drugs including CQ [12–14]. CQ and HCQ are both water-soluble drugs that are absorbed rapidly in the gut and have a long elimination half-life in the plasma of 900 and 1300 h respectively [15]. Multiple authors, including us, have reported in vitro and in vivo evidence of CQ-induced genotoxicity in the mammalian system. These drugs also possess immunomodulatory roles that have the potential to suppress the activation of the immune system in healthy individuals [16–18]. Considering the current situation, there is an urgent need for clinical studies to evaluate the clinical efficacy of HCQ and CQ as a prophylactic drug against COVID-19. We have not included the long-term side effects of these drugs since it is unlikely that prophylactic use of these drugs would be for a long time. Here in this review, we mainly aim to critically review and discuss the mutagenic, genotoxic, and immunomodulatory aspects of HCQ and CQ using the available literature.

## Review

### Mutagenic and genotoxic effect

**Fig. 1:**
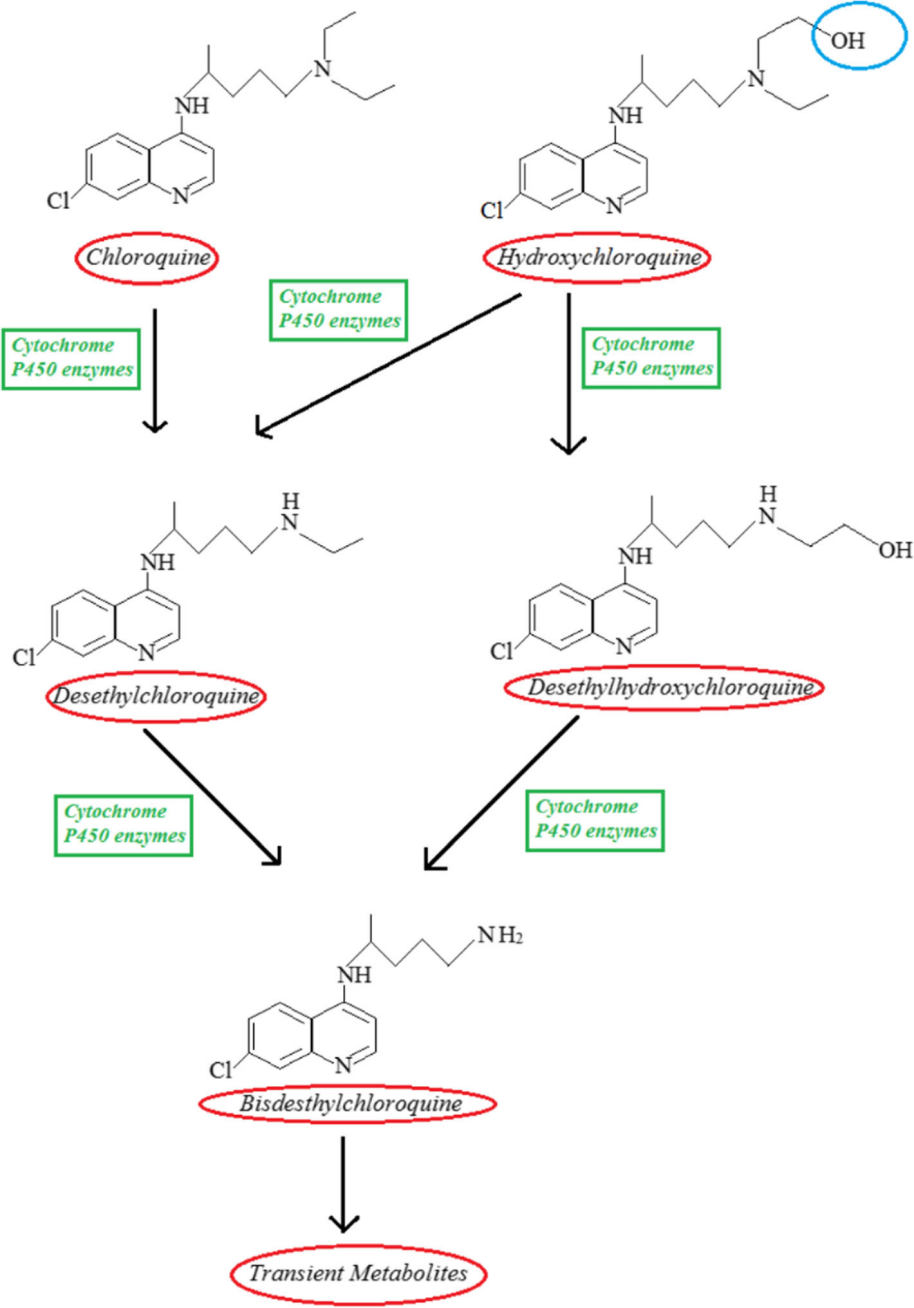
Comparison of structures and metabolism of Hydroxychloroquine (HCQ) and Chloroquine (CQ): Both HCQ and CQ have a very similar, basic, and a flat aromatic structure. The only difference is the presence of an additional hydroxyl (−OH) group in HCQ as highlighted by the blue oval in the representation. Dealkylation of HCQ and CQ are mediated by Cytochrome P450 enzymes in the liver. HCQ produces two first-stage metabolites instead of just one. The two metabolites are Desthylhydroxychloroquine and Desthylchloroquine. Desthylchloroquine is also the first-stage metabolite product of CQ. Both the first-stage metabolites are further metabolized to a common product, Bisdesthylchloroquine

Table 1 summarizes the mutagenic, genotoxic and carcinogenic effects of CQ in multiple test systems. It is interesting to note that there is almost no report on the mutagenic and genotoxic effects of HCQ both in vitro and in vivo. However, both HCQ and CQ have a very similar, flat aromatic core structure with a basic side chain. The only difference is the presence of an additional hydroxyl (−OH) group in HCQ. During metabolism, about 60% of both the drugs remain unchanged and about 40% of both the drugs are metabolized into two common metabolites desethylchloroquine and bisdesethylchloroquine by the action of the cytochrome P450 (CYP) enzymes in the liver [46]. Despite CQ being recognized as more toxic than HCQ, the tissue and plasma distribution of these two drugs were reported to be nearly identical when administered in equivalent dosage in humans [46]. Figure 1 shows the comparison of structures and metabolism of HCQ and CQ as described by Schrezenmeier and Dorner [47]. HCQ produces two first-stage metabolites instead of one. One being Desthylhyoxychloroquine and the other Desthylchloroquine. Desthylchloroquine is also the first-stage metabolite product of Chloroquine. Both the first-stage metabolites are further metabolized to a common product, Bisdesthylchloroquine [47].

**Table 1:** Mutagenic, genotoxic and carcinogenic effects of chloroquine in multiple test systems

Test system	Dose Used	Endpoints	Effects	References
**Bacterial strains**				
*Escherichia coli*	1 X 10^− 3^ M	DR	+	[19]
*Salmonella typhimurium* TA1537	100 and 250 μg/ml	MU	+	[20]
*Escherichia coli B/r*	50 μg/ml	MU	+	[21]
*Salmonella typhimurium* TA1537	10–10,000 μg/plate	MU	+	[22]
(*Salmonella typhimurium* TA1537, TA1538, TA98 and TA100	5–5000 μg/plate	MU	–	[23]
*Escherichia coli*	300–500 μg/ml	DR	+	[24]
*Salmonella typhimurium* TA1537 and TA97a	312–500 μg/plate	MU	_	[25]
*Drosophila melanogaster*	3–10 mM	SRL	+	[26]
*Salmonella typhimurium* TA100, TA1537, TA1538	5–10 μg/plate	MU	+	[27]
*Salmonella typhimurium* TA97 *Escherichia coli* EE97 and EE102	25–200 μg/ml	MU	+	[28]
*Salmonella typhimurium,* TA102 *E. coli* strains WP2, WP2hcr, WP6 and WP67	25–200 μg/ml	MU	–	[28]
*Salmonella typhimurium* TA97 *Escherichia coli* DG1669	25–500 μg/plate	MU	+	[29]
*Salmonella typhimurium* TA1537, TA1538, TA98 and TA100	100–600 μg/ml	MU	+	[30]
*Salmonella typhimurium* TA1977, TA1978	100–600 μg/ml	MU	–	[30]
*Salmonella typhimurium* TA97a and TA100, TA104	0.1–10,000 μg/plate	MU	±	[14]
*Salmonella typhimurium* TA98	192.5–6160 μg/plate	MU	±	[31]
*Salmonella typhimurium* TA98, TA100, TA97, TA1537	0–10,000 μg/plate; 250 μg/plate; 200 μg/ml	MU	+	[32]
*Salmonella typhimurium* TA1535, TA1977, TA102, TA104	10,000 μg/plate; 600 μg/plate 5000 μg/plate	MU	–	[32]
*Salmonella typhimurium* TA100	10–5000 μg/plate	MU	±	[33]
**In vitro** ***studies on mammalian systems***				
Rat liver cells	0.2 mM	DR	+	[34]
Human lymphocytes	15–100 μg/ml	CA	+	[35]
Chinese hamster ovary (CHO) cells	5–100 μM	MN SCE	+ +	[36]
Chinese hamster lung fibroblast (V79) & Rat hepatocyte cells (H4)	0.001–10 μg/ml	SCE	+	[27]
Chinese hamster lung fibroblast V79 cells	1–340 μM	MN	+	[37]
Rat liver cells	Full text not available	DD	+	[38]
Rat liver cells	0–1000 μM	DD	+	[39]
**In vivo** ***studies in multiple test systems***				
Rheumatoid patients	250 mg/day for 6 years	CA	+	[40]
Patients with aplastic anemia	Data not available. Patients took CQ for several months	CO^a^	+	[41]
Mouse bone marrow cells	12.5–100 mg/kg in single i.p. dose	CA SCE	+ +	[14]
Wistar rats	20 mg/kg orally thrice a week continued up to 400 days.	CO^b^	+	[42]
Mouse bone marrow cells	10–30 mg/kg in single i.p. dose and one sub acute i.p.dose 10 mg daily for 3 days.	MN CA	+ +	[43]
Egyptian Toad *Bufo*	Toads weighing 50 g gavaged 0.5 mg and after 6 h 0.1 mg and then 0.1 mg daily for 3 days	CO	+	[44]
Male Wistar rats	10 mg/kg i.p. for once a day for 7, 14 and 21 days	DD	+	[45]

+: Positive effect, ±: Weakly positive effect, −: Negative (non mutagenic) effect,

MU: Mutagenicity assay, CA: Chromosomal aberrations, SCE: Sister-Chromatid Exchange,

SRL: Sex linked recessive lethals. DD: DNA Damage, DR: Inhibition of DNA Repair,

MN: Micronuclei formation, CO: Carcinogenicity (Lymphosarcomas),

CO^a^: Carcinogenicity (Myeloblastic leukaemia), CO^b^: Co-Carcinogenic effects

While many authors have reported CQ to induce mutagenic effects in bacterial systems [20–22, 24, 27–30, 32], few authors had found weak or no mutagenic associations in certain bacterial strains [14, 23, 25, 28, 31–33]. Positive mutagenic effects (either weak positive or positive) reported by several authors showed that CQ is capable of inducing mutation in the Salmonella strains TA97, TA97a, TA153 and TA1538, which are used to detects the frameshift mutations. During the mutagenicity assay, most studies did not find any significant differences in the revertant numbers either with or without metabolic activation system (S9). This indicates that CQ is a direct-acting mutagen. In addition to Salmonella strains, CQ showed mutagenic effects in *Escherichia coli* EE97, EE102, DG1669 strains, and WP2, WP2hcr, WP6 and WP67 strains [21, 28–30]. CQ also can interact with DNA and produce an intercalated complex that may induced frameshift mutation by shifting the reading frame [48, 49]. This also indicates it’s DNA damage and inhibition of DNA repair potentials reported by several authors [19, 34, 38, 39, 45]. CQ is further reported to induce sex-linked recessive lethal mutation in *Drosophila melanogaster* [26]. These results indicate the mutagenic potentials of CQ in bacterial systems.

In a broad genotoxic review on several antimalarial drugs, cumulative pieces of evidence pointed out that CQ is also a genotoxic drug [50]. Our study on CQ has demonstrated genotoxic effect as measured by chromosomal aberrations (CA), sister chromatid exchange (SCE), and micronuclei (MN) formations in vivo in mice [14]. These results are in agreement with several other authors who have reported CQ to be a genotoxic drug in both in vitro and in vivo systems [35–37, 40, 43]. CQ has also been reported to induce oxidative stress in animal models [51]. For instance, when CQ administered intraperitoneally in rats, it induced DNA breaks in the kidney within 1 to 2 weeks and in the liver within 2 to 3 weeks [45]. Chromosomal Aberrations (CA) have also been long considered to be a predictor for cancer. Rossner et al., [52] reported a strong association between increased frequencies of CA in cells and an increased risk for cancer using a cohort of 11,834 subjects. CA along with other genotoxic effects like SCE and MN as reported here, suggests that long-term use of CQ can induce significant chromosomal damages which may lead to an increased risk of cancer in humans.

CQ is not considered carcinogenic due to inadequate evidence pointing to carcinogenicity in humans. Yet, a well-controlled study by El-Mofty et al., [44] in Egyptian toad showed that separately CQ and primaquine can induced tumor formation in 14 and 19% of the toads respectively. They further showed that CQ and primaquine when used together the incidence of tumor rose to 23.5% [44]. This type of co-tumorigenic effect of CQ was also observed in another study by Reyes et al., [42] where CQ promoted the carcinogenic effect of a drug called ethynitrosourea on ependymal cells of rodents in vivo*.* The only report of CQ induced Aplasis and leukemia was observed in a patient treated with long term CQ therapy [41]. Brambilla and Martelli, [53] showed that *N*-nitroso compounds, which are capable of inducing genotoxic effects and tumor formation in animal models, can be formed in the gastric environment when CQ is used with nitrite drugs. So, the genotoxic-carcinogenic effect may be induced when nitrite drugs are taken along with amine drugs like CQ and HCQ. Results presented in the Table 1 indicate that CQ is mutagenic and genotoxic drug in both bacterial systems, and in vitro and in vivo on mammalian systems.

### Immunomodulatory effects

**Fig. 2:**
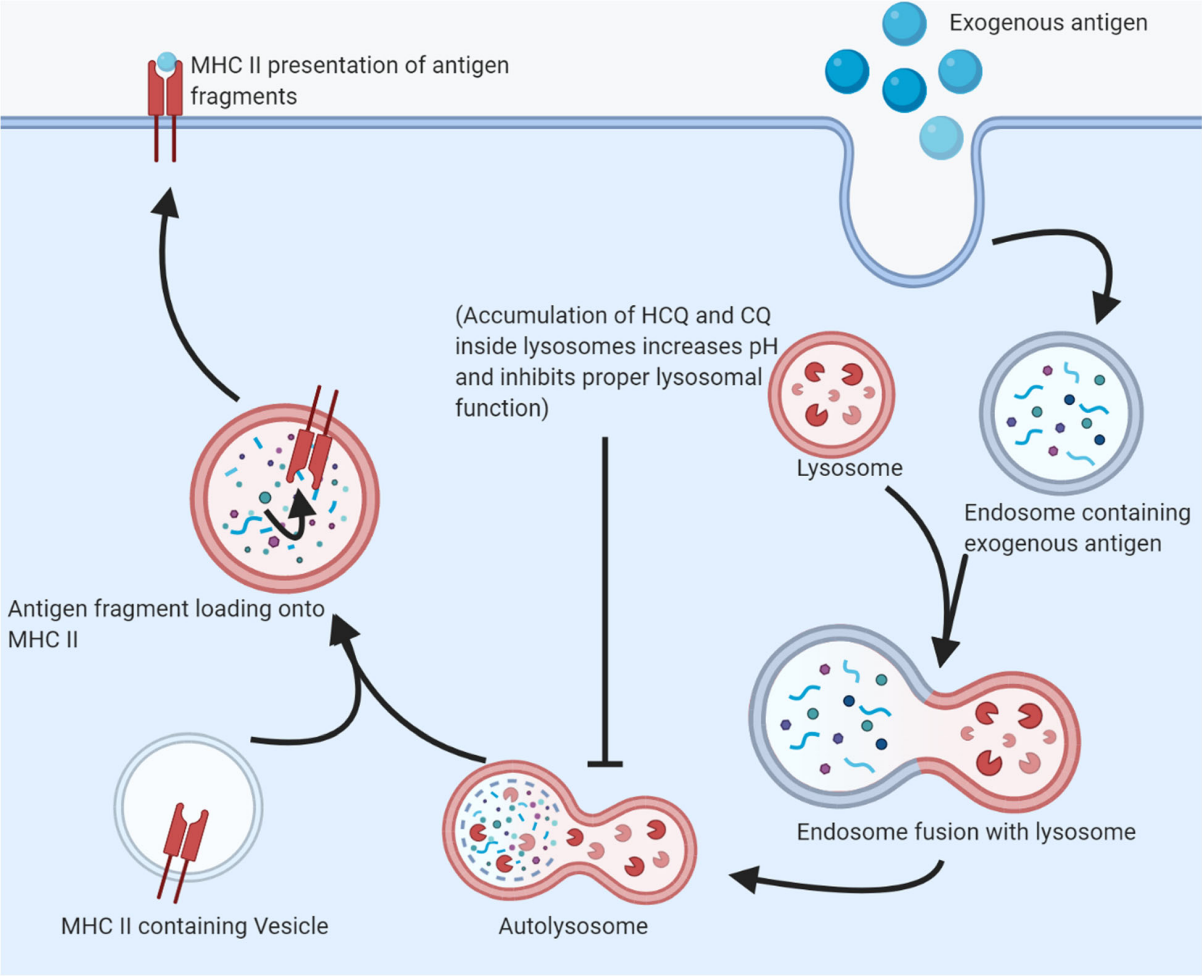
Intervention of HCQ and CQ in the exogenous pathway of antigen presentation: Exogenous antigens that are taken up either by endocytosis or phagocytosis requires fusion with lysosomes for the process of degradation. Finally, the degraded antigen fragments are loaded onto the MHC class II molecules for antigen presentation. Lysosomotropic drugs like HCQ and CQ accumulate inside the lysosome and these drugs potentially increase the pH inside and disrupt lysosomal functions

Table 2 summarizes the available reports on the immunomodulatory aspects of HCQ and CQ in multiple test systems. Both HCQ and CQ has been reported to inhibit the activation of the immune system in many ways. Lysosomotropic drugs (like HCQ and CQ) can accumulate inside lysosomes and being basic they can increase the pH inside the lysosomes and prevent its normal functions [63, 64]. These drugs can also cause lysosomal membrane destabilization and thus the release of lysosomal contents and enzymes inside the cells [63]. Lysosomes have an essential role in the exogenous (lysosomal) pathways of antigen presentation and therefore proper lysosome functions are essential for MHC class II antigen processing and presentation. The intervention of HCQ and CQ in the exogenous pathway of antigen presentation has been presented in Fig. 2 [64].

**Table 2:** Immunomodulatory effects of hydroxychloroquine (HCQ) and chloroquine (CQ) in multiple test systems

Test systems	Drugs	Dose Range	Endpoints	Effects	References
Human lymphocytes in vitro (PBMC)	CQ	0–25 μg/ml	Inhibition of generation of immunoglobulin-secreting cells and IL-1 secretion	+	[54]
Systemic Lupus Erythematosus Patients	HCQ	400 mg/day for 12 weeks	Decreased levels of cytokines (IL-6), soluble CD8 and soluble IL-2 receptors	+	[55]
T-cell line	HCQ	0–100 μM	Inhibits calcium signals in T cells	+	[18]
PBMC from healthy donors	CQ	0–10 μM	Inhibition of RNA- mediated TLR7 activation; inhibition of IFN-α	+	[56]
PBMC and monocytic U-937 and THP-1 cells in vitro	CQ	0–100 μM	Inhibition of cytokine (TNF-α, IL-1β and IL-6) production.	+	[57]
Macrophage-like RAW264 cells in vitro	CQ	0–100 μM	Inhibition of Toll-like Receptor (TLR) signaling pathways	+	[58]
Systemic Lupus Erythematosus Patients, Human embryonic kidney cells	CQ	5–10 μg/ml	Inhibits activation of endosomal TLRs	+	[4]
Systemic Lupus Erythematosus Patients	HCQ	200–400 mg/day	Impaired IFN-α and TNF-α production	+	[5]
Human lymphocytes in vitro (PBMC)	HCQ	0–20 nmol/L	Inhibition of autophagy, and induction of apoptosis of memory T-cells	+	[59]
MonoMac1 (MM1) cells, human monocytes	HCQ	0–100 μM	Inhibits pro-inflammatory signaling pathways, prevents the induction of endosomal NADPH oxidase	+	[60]
Systemic Lupus Erythematosus Patients and on Jurkat Cell	HCQ	Patients blood samples and in Jurkat cell 600–6000 ng/ml	Inhibition of NFAT signaling in activated T cells and CD154 expression in CD4^+^T cells	+	[17]
A549Pt and A549cisR non-small-cell lung cancer cell lines	CQ	25–100 μM	Lysosomal membrane destabilization and autophagy	+	[16]
PBMC from healthy volunteer	HCQ	Data not available	BlocksTLR9 ligation in class-switched memory B cells, suppress mTORC1 signaling.	+	[61]
Rosetta BL21 pLysS cells	HCQ and CQ	1–4 μM	Blockade of c-GAS-DNA interaction	+	[62]

+: positive effects, CQ: Chloroquine, HCQ: Hydroxychloroquine, IL: Interleukin,

TLR: Toll-like receptor, PBMC: Peripheral blood mononuclear cells,

TNF: Tumor necrosis factor; cGAS: Cyclic GMP-AMP synthase

**Fig. 3:**
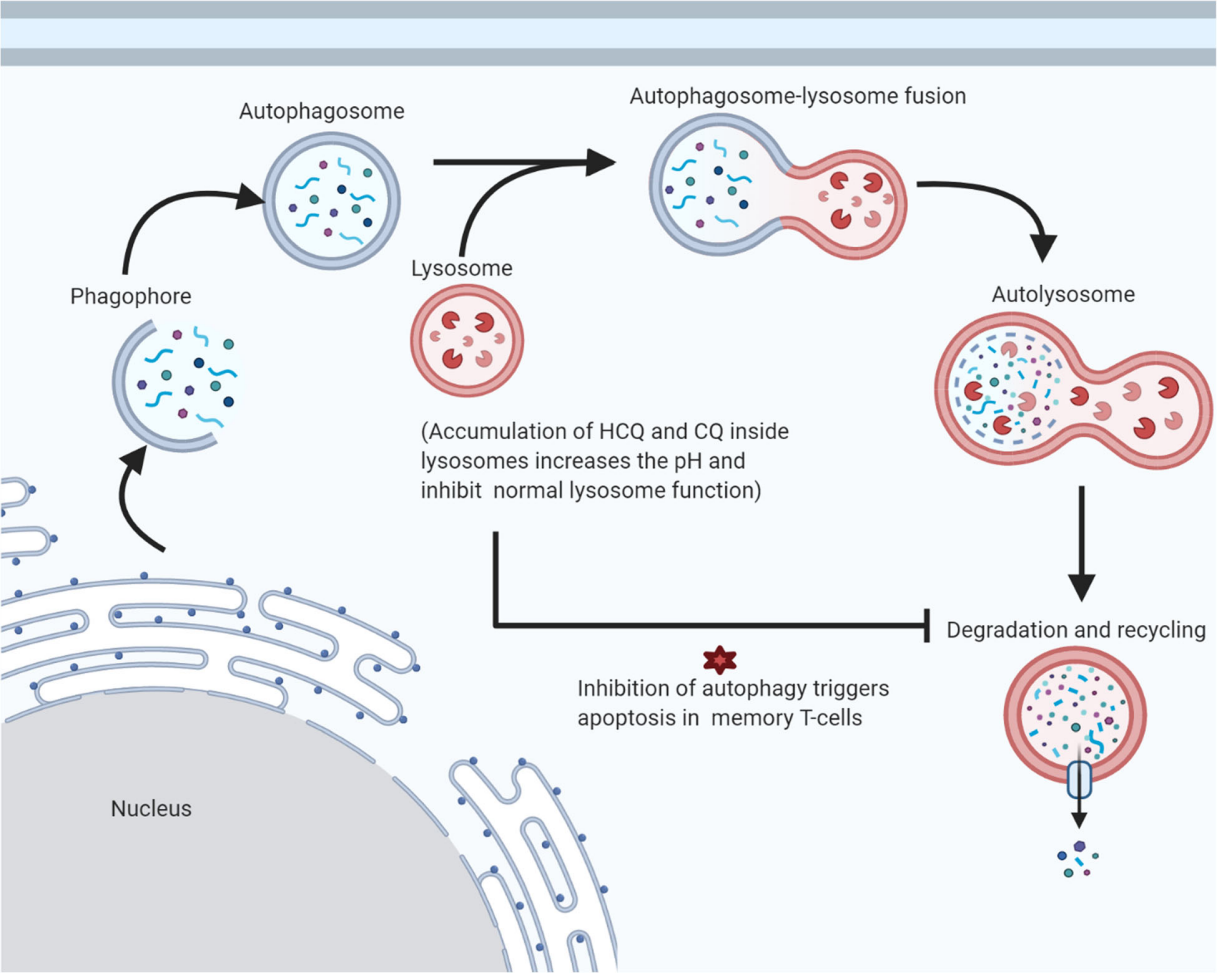
Inhibition of autophagy by HCQ and CQ triggers apoptosis: Autophagy is often utilized by cells as a survival mechanism to prevent apoptosis. Normal autophagy process includes the fusion of autophagosomes with the lysosome, followed by the degradation and recycling of the internal components like damaged organelles. Accumulation of HCQ and CQ increases the pH inside the lysosomes and prevents the normal autophagy process. This prevention of autophagy triggers apoptosis in memory T-cells and this is one of the fundamental mechanisms of immunomodulation by HCQ and CQ. Also, not shown in the diagram, inhibition of autophagy can also reduce antigen presentation via MHC class II molecules

Autophagy is an implied concept in immunity development. Besides the degradation and recycling of endogenous substrates, the process of autophagy is a key mechanism used by cells to tackle intracellular pathogens [65]. HCQ and CQ both can potentially inhibit the normal autophagy processes. Autophagosomes require fusion with the lysosomes to start the process of degradation. The increased pH of lysosomes, due to HCQ and CQ intervention, inhibits the maturation of the autolysosome. The inhibition of autophagy has been further linked to the induction of apoptosis of memory T-cells, which is the basis of the mechanism of immunomodulation by these drugs in several autoimmune diseases [59, 66]. Mechanism of the inhibition of autophagy by HCQ and CQ which triggers apoptosis has been presented in Fig. 3 [59]. HCQ can further block endosomal activation of NADPH oxidase (NOX2) that normally generates the reactive oxygen species and involved in the proinflammatory response of the immune system [60]. With a decreased activity of NADPH oxidase, cells can phagocyte pathogens but can’t degrade them inside the phagocytic vesicle.

**Fig. 4:**
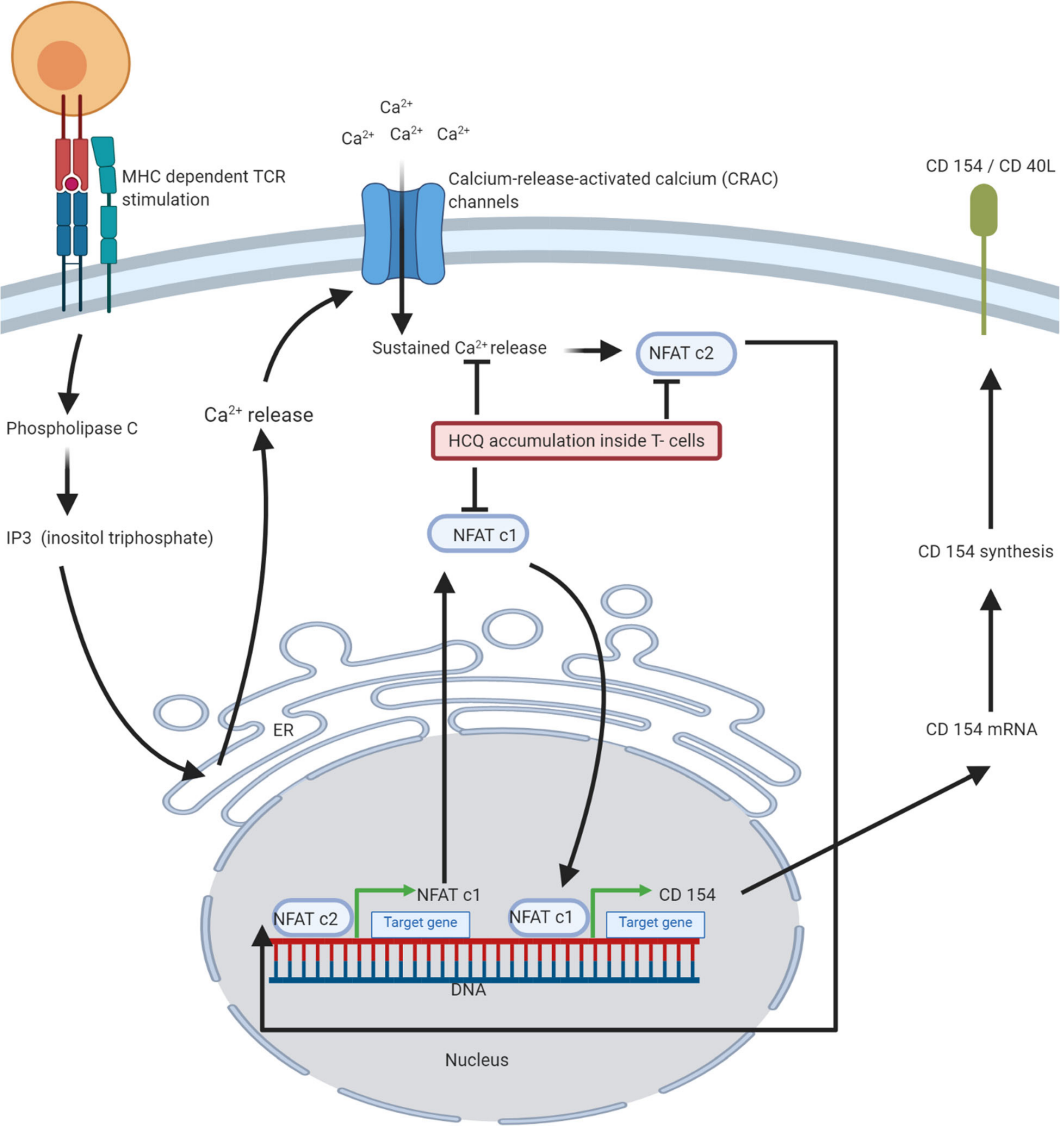
HCQ interference in the T-cell activation pathway and transcription of CD 154: When T-cell receptor (TCR) is stimulated by antigen via MHC, a series of events leads to the activation of the Phospholipase C, which then generates Inositol triphosphate (IP3). IP3 induces the release of calcium from the endoplasmic reticulum (ER). Calcium acts as a secondary messenger to activate Calcium-release-activated Calcium channel (CRAC) for a steady influx of extracellular calcium. Intracellular calcium binds to calmodulin and activates the phosphatase calcineurin (not shown in the diagram). Calcineurin dephosphorylates and activates transcription factor NFATc2. NFATc2 migrates the nucleus and triggers the transcription of NFATc1. NFATc1 mRNA is exported outside the nucleus where de novo synthesis of transcription factor NFATc1 occurs. NFATc1 then migrates back into the nucleus to triggers the transcription of CD 154. HCQ can potentially interfere with intracellular calcium signaling and prevent dephosphorylation and activation of the transcription factor NFAT. This is one of the mechanisms by which HCQ interfere in T-cell activation and CD 154 transcription

HCQ can also function as an immunosuppressant by blocking steps in the T-cell activation pathway. HCQ has been shown to inhibit transcription factor NFAT (Nuclear Factor of Activated T-cells) upon T-cell activation in vivo and block expression of co-stimulatory ligand CD154 i.e. CD 40 L, which initiates T-cell dependent B-cell proliferation and antibody formation [17]. The probable mechanism of the interference in the T-cell activation by HCQ has been presented in Fig. 4 [17, 18]. HCQ intervention can further down-regulate the CD69 marker in healthy controls by inhibiting calcium mobilization and dephosphorylation of NFAT [18]. CD69 functions as a costimulatory molecule for T-cell activation and proliferation. The same study showed that B-cell antigen receptor calcium signaling was also reduced by HCQ intervention [18]*.*

Patients on antimalarial drugs like HCQ and CQ have lower levels of IL-6, soluble CD8, and IL-2 receptors which is beneficial for those suffering from autoimmune diseases like SLE and RA [55]. Studies have shown that CQ, at doses that are expected in the serum of treated patients, was able to interfere with mitogenic-response of monocytes and this diminished mitogenic response was determined to be irreversible [54]. Furthermore, CQ inhibited the generation of Immunoglobulin-secreting cells by preventing the capacity of monocytes to secrete factors like IL-1 [54]. Normal secretion of TNF-α, IL-1b, and IL-6 by monocytes or macrophages was also inhibited by CQ [57].

**Fig. 5:**
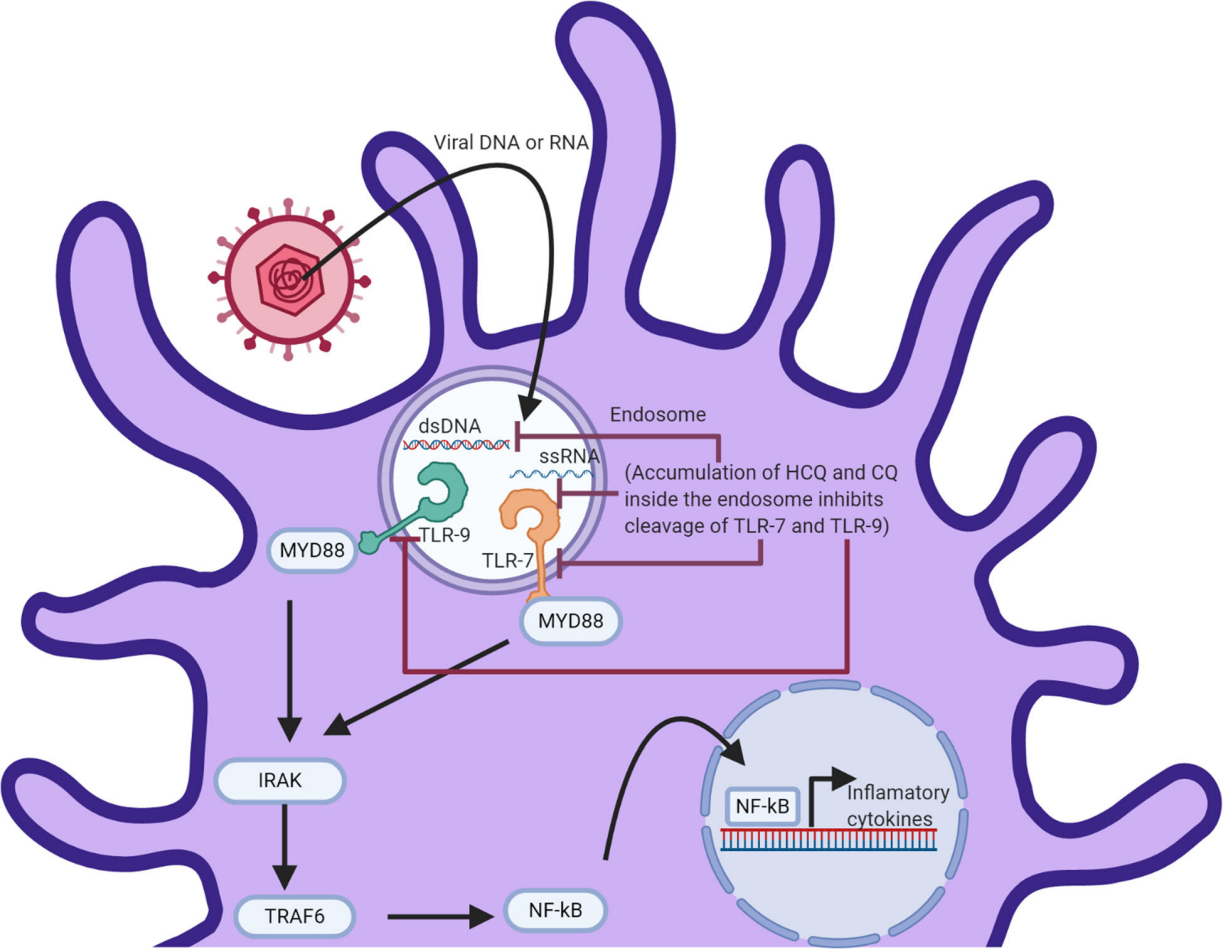
Endosomal TLR 7 and 9 inhibition by HCQ and CQ: Mammalian Toll-like receptors (TLR) 7 and 9 initiate immune response when it encounters microbial nucleic acids (only shown here is a viral particle). The ectodomain of TLR 7 and 9 are cleaved in the endolysosome (not shown) which then recruits MYD88, followed by the activation of IRAK, TRAF6, and NF-kB. NF-kB then migrates to the nucleus and triggers the transcription of inflammatory cytokines. Both HCQ and CQ can increase the pH of the endolysosome and interfere with the TLR 9 and TLR 7 cleavage and processing. Furthermore, HCQ and CQ can directly bind to the microbial nucleic acids and prevent TLR-ligand interaction

HCQ and CQ can potentially inhibit Toll-like receptor (TLR) signaling of TLR7 and TLR9 in Antigen Presenting Cells (APCs) including dendritic cells, macrophages and B-cells. Normally, upon activation of by nucleic acids, endolysosomal TLR7 and TLR9 are cleaved which in turn activates MyD88 and triggers an innate immune response in the downstream cascade. This proteolytic cleavage is inhibited by the changes in the endosomal pH as a result of HCQ and CQ interference [58]. The mechanism of the inhibition of endosomal TLR 7 and TLR9 by HCQ and CQ has been presented in Fig. 5 [58]. HCQ and CQ have further been shown to directly bind to nucleic acid, inhibiting TLR-nucleic acid interaction and preventing TLR9 activation [4], and CQ has been shown to inhibit RNA-mediated TLR7 activation [56]. HCQ treatment in vivo caused a significant reduction of the production of INF-α and TNF-α by the plasmacytoid Dendritic cells by suppressing the activation of TLR7 and TLR9 [5]. Innate TLR signaling leads to the production of cytokines such as IL-6, TNF-α, and IL-1 that eventually triggers the adaptive immune response. At clinical concentration, HCQ can efficiently block TLR9 ligation and have an inhibitory effect on class-switched memory B-cells [61].

**Fig. 6:**
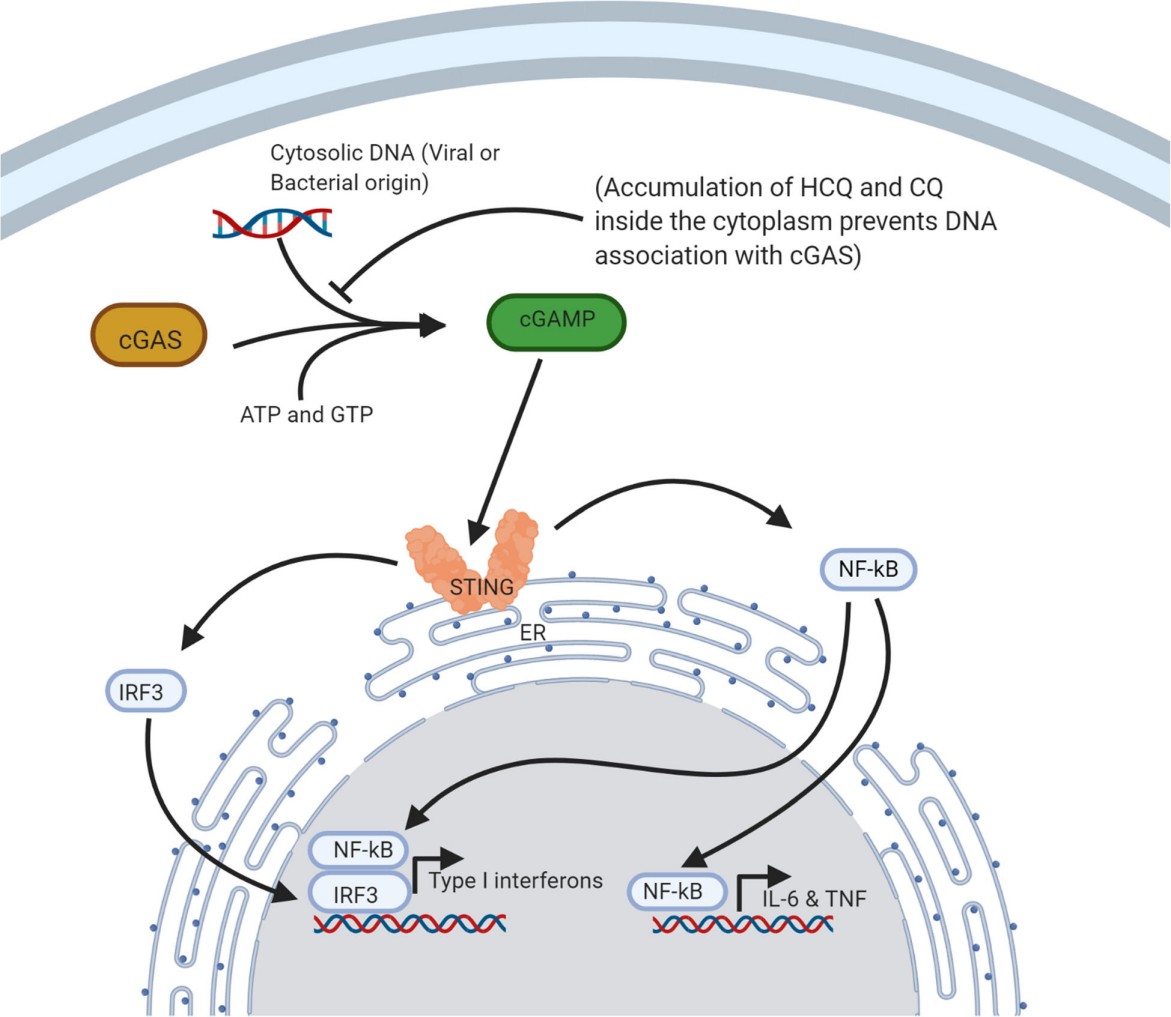
cGAS-STING Pathway inhibition by HCQ and CQ: DNA sensor cGAS (cyclic GMP-AMP synthase) can recognize DNA of viral/bacterial origin and synthesize a dinucleotide molecule, cGAMP (2′, 5′-cyclic GMP-AMP dinucleotide). Newly synthesized cGAMP can activate an endoplasmic reticulum (ER)-associated protein STING (Stimulator of interferon genes). STING potentially activates the transcription factors IRF3 and NF-kB, which can then migrate to the nucleus and lead to the transcription of type I IFNs and cytokines. Although HCQ/CQ doesn’t directly bind to the active site of cGAS, these molecules can occupy the minor grooves of DNA molecule and prevent association with cGAS

The cGAS-STING is another pathway that is involved in the type I interferon response of innate immunity. Cyclic GMP-AMP synthase (cGAS) is a nucleotidyltransferase that is activated to cGAMP (2′,5′ –cyclic GMP-AMP dinucleotide) when dsDNA, usually from a viral or bacterial origin, binds to it. cGMAP then activates an endoplasmic reticulum membrane-associated protein known as the STING (stimulator of interferon genes). Activation of STING leads to the activation of the transcription factor IRF3 and NF-kB, which then can migrate to the nucleus to activate the Type I IFNs and cytokines [67]. Evidence suggests one-way HCQ and CQ can achieve the immunomodulatory effect is because of its ability to suppress the activation of this pathway by inhibiting ligand binding [62]. The mechanism of the inhibition of cGAS-STING Pathway by HCQ and CQ has been presented in Fig. 6 [62].

Given all the above mechanisms, it is now clear that these drugs can effectively modulate cellular signaling. Sometimes, this can be helpful but only when used by patients infected with COVID-19. For instance, COVID-19 patients have usually higher levels of inflammatory cytokines which ultimately result in collateral damage to the host tissues. The use of HCQ and CQ has been reported to reduce the overproduction of IL-1B, IL-6, and Granulocyte-colony stimulating factor (GM-CSF). The reduction in endosomal acidification as a result of HCQ and CQ accumulation is believed to halt or reduce the disruption of viral particles and thus the release of infectious nucleic acid. The ACE2 receptors of the lungs are required for SARS-CoV2 entry into the host cells. Glycosylation of ACE2 receptors is required for the translocation of ACE2 receptors to the cell membrane. HCQ has also been shown to reduce this glycosylation step [68]. These studies have together motivated healthcare workers to use these drugs as a prophylactic, post-exposure prophylactic, and as a curative drug. Unfortunately, these results were only successful in vitro. As we will discuss later, large clinical trials have found no evidence of the benefit of using these drugs.

### Genetic variation leading to a difference in the metabolism of HCQ and CQ

It is important to remember the metabolism of these drugs in vivo. Both HCQ and CQ are metabolized in the liver by an enzyme known as cytochrome P450 (CYP) and the gene expression of this enzyme varies between different individuals as a result of the difference in nucleotide polymorphisms [69]. Furthermore, these polymorphisms have been associated with the formation of unstable enzymes and thus a decreased in its activity [70]. Some ethnicities have a complete absence of certain functional CYP enzymes like the CYP 2D6, which is one of the important CYP enzymes that is actively involved in the metabolism of these drugs [69]. An Individual’s P450 polymorphisms should be taken into account when considering to prescribe HCQ since there is a clear association between different polymorphisms in CYP 2D6 and the blood concentration of HCQ in SLE patients [69]. These polymorphisms have also been linked to the toxic accumulation of these drugs in the blood of the patients treated with HCQ and CQ [71].

### Latest clinical findings

To date, there is still no evidence that points to any benefit of using these drugs against the COVID-19 as a prophylactic. Moreover, the potentials of these drugs as a post-exposure prophylactic or curative drug has also been called into question. Recently, the Food and Drug Administration (FDA) of the United States (US) has revoked their Emergency Use Authorization (EUA) for emergency use of these drugs [72], and the World Health Organization (WHO) [73], along with National Institute of Health (NIH) [74] in the US had also stopped conducting further clinical studies because of sufficient evidence that these drugs provided no benefit.

Initially, a few in vitro evidence and small studies suggested that these medications might be helpful, however, large scale studies have found no such significance. The first clinical evidence of a positive result stems from a small study in China where 100 subjects treated with CQ were found to have a superior benefit over the control group suffering from COVID-19 pneumonia [75]. Another small study in France with only 26 subjects, who received HCQ, showed a significant reduction in the viral load compared to the control group [76]. The time to clinical recovery was also shown to be reduced in those under HCQ treatment in a small randomized trial of just 62 COVID-19 patients [77]. Clearly, the small sample size was a major drawback in these studies. However, larger clinical randomized controlled trial has now been conducted and results have provided us with a different outcome. For instance, a recent study in Brazil with 504 confirmed COVID-19 patients, HCQ treatment had no significant benefit over no treatment group [78]. Furthermore, Chloroquine at high doses (600 mg, twice daily) for 10 consecutive days was associated with higher lethality and is now not recommended for critically ill patients [79]. A large randomized clinical study investigating the post-exposure prophylactic role of HCQ found that a higher dose of HCQ did not prevent infection when treatment was initiated within 4 days after exposure [80]. Another study showed us that in 368 COVID-19 patients those taking HCQ only or HCQ plus Azithromycin had a death rate of 27.8 and 22.1% respectively when compared to 11.4% in the no-treatment group [81].

## Conclusions

Most pathogenic microorganisms that infect humans are tackled satisfactorily by the innate and adaptive immune system of our body. Healthy humans have an extraordinary capability to fight off infections caused by pathogenic microorganisms. The activation of innate immunity initiates the first line of defense until the more specific adaptive immunity develops. Existing literature suggests that HCQ and CQ can potentially interfere with both innate and adaptive immune responses in multiple ways. Here in this review, we have highlighted the known pathways where HCQ and CQ can intervene to achieve its immunomodulatory effects and also provided systematic diagrams for a better understanding of the affected pathways.

Furthermore, CQ has been recognized to induce genomic instability by inducing mutagenic, genotoxic, and DNA damages both in vivo and in vitro systems. Most authors, including us, found weak to moderately strong mutagenic effects in different Salmonella strains routinely used for mutagenicity screening of drugs and chemicals. Moreover, almost all authors who worked on genotoxicity assays have reported positive genotoxic effects of CQ in multiple test systems. This indicates that CQ is a mutagenic and genotoxic drug. However, with a lack of sufficient studies on the genetic toxicology of HCQ both in vitro and in vivo, the mutagenic and genotoxic effects of HCQ remains inconclusive.

Current evidence cumulatively demonstrates that both HCQ and CQ are not effective against the COVID-19 infection either as post-exposure prophylaxis or as a curative drug. No studies on the prophylactic role of these drugs have been evaluated to date. The world is going through tremendous turmoil because of the COVID-19 pandemic. Thus, we do recognize the importance of optimism and the implementation of any advancement in science during this emergency. Thus, the use of these drugs post-infection might be useful, but this discussion is beyond the scope of this review. Here, we are mainly concerned with the use of these drugs by healthy individuals as a prophylactic without any evidence. So, without any clinical or in vivo evidence, current literature suggests that healthy individuals should refrain from the use of these drugs as prophylactics until further investigation.

## Data Availability

Not applicable.

## **Abbreviations**

CQ: Chloroquine
HCQ: Hydroxychloroquine
COVID-19: novel Severe Acute Respiratory Syndrome Corona Virus 2
RA: Rheumatoid Arthritis
SLE: Systemic Lupus Erythematosus
CYP: cytochrome P450
S9: Metabolic activation system
+: Positive effect
±: Weakly positive effect
-: Negative (non mutagenic) effect
MU: Mutagenicity assay
CA: Chromosomal aberrations
SCE: Sister-Chromatid Exchang
SRL: Sex linked recessive lethals
DD: DNA Damage
DR: Inhibition of DNA Repair
MN: Micronuclei formation
CO: Carcinogenicity
IL: Interleukin
TLR: Toll-like receptor
PBMC: Peripheral blood mononuclear cells
TNF: Tumor necrosis factor
NFAT: Nuclear Factor of Activated T-cells
cGAS: Cyclic GMP-AMP synthase
STING: Stimulator of interferon genes
ACE2: Angiotensin-converting enzyme 2
GM-CSF: Granulocyte-colony stimulating factor
